# Animal movements in the Kenya Rift and evidence for the earliest ambush hunting by hominins

**DOI:** 10.1038/srep14011

**Published:** 2015-09-15

**Authors:** Simon Kübler, Peter Owenga, Sally C. Reynolds, Stephen M. Rucina, Geoffrey C. P. King

**Affiliations:** 1 Department of Earth and Environmental Sciences, Ludwig-Maximilians-University Munich, Germany; 2 Kenyan National Agricultural Research Laboratory (KALRO-Kabete) Nairobi, Kenya; 3 Institute for Studies of Landscape and Human Evolution, Faculty of Science and Technology, Bournemouth University, Bournemouth, UK, BH12 5BB; 4 Department of Earth Sciences, National Museums of Kenya, Nairobi, Kenya; 5 Labo Tectonique, Institute de Physique du Globe de Paris, Paris, France

## Abstract

Animal movements in the Kenya Rift Valley today are influenced by a combination of topography and trace nutrient distribution. These patterns would have been the same in the past when hominins inhabited the area. We use this approach to create a landscape reconstruction of Olorgesailie, a key site in the East African Rift with abundant evidence of large-mammal butchery between ~1.2 and ~0.5 Ma BP. The site location in relation to limited animal routes through the area show that hominins were aware of animal movements and used the location for ambush hunting during the Lower to Middle Pleistocene. These features explain the importance of Olorgesailie as a preferred location of repeated hominin activity through multiple changes in climate and local environmental conditions, and provide insights into the cognitive and hunting abilities of *Homo erectus* while indicating that their activities at the site were aimed at hunting, rather than scavenging.

Olorgesailie in the southern Kenya Rift is famous for its unusual abundance of hominin artefacts, fossil mammals and palaeoenvironmental indicators, preserved in sediments spanning ~1.2 to <0.5 Ma and has been the subject of wide-ranging and intensive studies on hominins and their archeology^1^,^2^,^3^,^4^, butchery behaviour^5^ and taphonomy^6^, dating and palaeoenvironmental and climatic changes and their impact on human evolution^4^,^7^,^8^,^9^,^10^,^11^,^12^,^13^. The rich cultural assemblage is predominantly Acheulean and is found in several layers associated with a palaeolake environment (Supplementary Material Section-5, and Figure S11), alternating between slightly saline, fresh and wetland phases^4^,^12^,^13^. The origin of artefact accumulation at the site has been the matter of debate ranging from *in-situ* deposition by hominins to minor fluvial reorganisation^1^,^14^. One cranial specimen of *Homo* has been recovered^6^ and the site preserves evidence of butchery by hominins^1^,^3^. The number of butchered animals, combined with the types of prey, suggest that the hominins were skilful hunters, although their methods have not been previously understood, nor have remains of weapon technology of any kind been discovered^6^,^13^. The first documented record of close encounter hunting of dangerous animals via wooden spears is from the Middle Palaeolithic of Europe, around 400 ka, at Schöningen, Germany^15^.

The site lies in the centre of the rift floor which is ~60 km wide with many sub-parallel, nearly vertical, fault escarpments^16^ constraining east–west movement of large animals and humans (Figs 1 and 2). A large area of the rift floor is covered by trachyte flows that resist erosion while other volcanic rocks including basalts and tuffs are associated with two volcanic edifices (Mts Olorgesailie and Esayeti). Small areas are infilled with sediments carried from the north and the edges of the rift^17^. Uplift and back tilting prevents the entry of sediments from outside the main rift. Saline Lake Magadi and associated sediments fill the lowest point in the region (Supplementary Material Section-1).

The fossil-rich Olorgesailie sediments deposited in the palaeolake are exposed along a 5–6 km escarpment and preserve transitions in basin hydrology and sedimentary regime that reflect shifts between terrestrial and shallow lake environments, presumably due to a combination of climate changes, earthquakes, and volcanic activity^10^,^12^,^13^. Here we reconstruct the wider topographic setting in which this palaeolake was situated, and examine the edaphic properties of soils (edaphics) which result on the different underlying geology (regolith), to provide a new way to understand the constraints on animals movements, both today and in the past. With these tools we identify the unique features of the site region that help explain why it repeatedly attracted hominin tool making and butchery activity over extended periods of time. The landscape around Olorgesailie today (Fig. 3a) is significantly different from when the hominins were present between 1.2 and 0.5 Ma. The two key processes responsible for landscape modification are: (a) tectonic motion on two north-south trending normal faults resulting in westward tilting of the Legemunge lakebeds. Fault motion post-dates the deposition of the Legemunge beds containing the hominin artefacts, as they as they were originally deposited horizontally and (b) caldera collapse of the northwestern Olorgesailie edifice resulting in draining of the palaeolake (Fig. 3, Supplementary Material Section-2). The geometry of the landscape past and present is defined by its tectonic structure, allowing reconstruction of palaeo-morphology to be attempted by modelling fault displacements. By removing the effects of fault motion and making corrections for erosion and deposition of sediment we have created a palaeo-DEM of the landscape as it appeared during the period when hominins were present (Fig. 3b–d and Supplementary Material Sections-1 and -2). The reconstruction allows us to consider how the movements of animals, such as large ungulates species, were constrained by the lake and topography.

Present-day wild and domestic animals move between the patchily distributed resources in the Kenya Rift^18^,^19^. Movement is controlled by the edaphic quality of soils, determined by the presence or absence of vital trace elements and macronutrients (e.g., Ca, Mg, P, Na, Cu and Co). If these are lacking, grazing and browsing animals will risk predation to seek out better fodder^20^,^21^. To extrapolate edaphic properties of modern soils to Pleistocene soils depends on establishing the relationship between the mineral properties of the parent bedrock and/or sediments and the overlying soils and vegetation^20^. Soils in the study area are developed on trachyte, volcanic bedrock and on fluvial and lacustrine sediments^17^ and we summarise modern analyses of macronutrients and trace elements from a representative sample of lithological and sedimentary units (Fig. 4, Supplementary Material Section-3 and -4). Trachytes and poor soils dominate the region while richer soils develop on some other volcanic rocks and on sediments brought by rivers from the north and east.

Calcium and to some extent magnesium and phosphorus are generally limited (Fig. 4 and Supplementary Material Section-3), and prolonged deficiency results in severe skeletal diseases like osteoporosis, retarded growth and rickets^22^,^23^,^24^. Calcium levels are only high in soils on palaeolake beds and river sediments, confirmed by measurements of plant tissue samples from the lakebeds and flood plain deposits close to the Olorgesailie site (Fig. 3b) and are generally low elsewhere^25^,^26^. Soils on trachytes appear to be always poor, and samples we have taken from other parts of the Kenya rift emphasise the general edaphic inadequacy of these soils (Supplementary Material Section-4).

To corroborate these results and identify the location of good grazing/browsing areas we interviewed the heads of all pastoral (Maasai) families in the region. Their herding experience corroborates to our edaphic analysis of favoured and disfavoured grazing areas (Supplementary Material Section-4).

We consider that the distribution of palaeofauna will have been subject to the topographic and nutritional constraints outlined. The palaeofauna of Olorgesailie (Supplementary Material Section-5) include three extinct and dangerous species that were butchered by hominins: the giant gelada (*Theropithecus oswaldi*), elephant (*Elephas recki*) and hippopotamus (*Hippopotamus gorgops*)^1^,^8^. Equids and grazing bovids dominate the faunal community. Fault barriers (Figs 2a,b and 3b) that would have been present when these communities roamed the area indicates the likely constraints that this topography would have imposed on animal movement, especially the ungulates. A tectonically active region like the immediate Olorgesailie region is insensitive to climate change^27^,^28^ and would have been attractive to animals even during drier periods. The total grazing area was limited, however, so large herbivores would have visited the region only during the dry season and moved to the rift flanks, and more extensive grazing and browsing at other times, making little or no use of the trachyte lands through which they passed (Fig. 3b indicates possible routes). Such regions away from Lake Magadi would have been climatically sensitive^27^. The extinct gelada baboons could have remained throughout the year exploiting the steep cliffs and the rough terrain of the volcanic edifice and vegetation otherwise inaccessible to large herbivores. The only predator preserved is the spotted hyena (*Crocuta crocuta*), and the number of individuals found is low. A relative lack of more dangerous predators, such as lions, would have added to the attraction for *Homo*. However, low predator numbers means that hominins would not have been able to reliably scavenge kills of other predators and must have been competent in close-encounter kills of larger-bodied and dangerous animals.

We conclude that the Olorgesailie site was attractive to hominins for six independent reasons: (a) it was located close to a lake providing reliable drinking water; (b) workable stone^1^ was available locally; (c) it controlled the only route allowing movement of large animals between east and west (d) nearby high-elevation areas provided possible lookout points; (e) the local region has excellent edaphics in a wider region that is highly deficient; and (f) there is a relative lack of dangerous predators.

The landscape complexity and the patchy nature of healthy grazing and browsing provide a way to understand how Pleistocene animals moved through the region and explain the hominin hunting and butchery activities at the Olorgesailie site over periods of significant climate fluctuations^10^. We argue that this is based on the strategic use of the landscape to gain advantage over large prey. The hominin exploitation of the Olorgesailie Site is explained by the predictable patterns of animal movement that would have allowed for ambush hunting. Animals remember danger and vary their routes where possible^29^. In the Olorgesailie region, and the south Kenya rift, alternative routes are absent, and we are led to a seemingly counterintuitive conclusion that *Homo* exploited this part of the Kenya rift not because it was generally “good” for herbivores, but because it was generally “bad”, and constrained their movements to predictable pathways which allowed them to be exploited by early hunters. This, in conjunction with raw material availability, low predator numbers and suitable ambush positions explains the repeated attraction of the site within the wider region.

This study combines two new approaches to explain the hominin presence in the region and provides new insights into the significance of the location of the Olorgesailie site. Our preliminary results around Kariandusi and Baringo indicate that they share similar features, and that the methods we describe can be applied more widely in the East African Rift and that hominins at these other sites were practising similar techniques of ambush hunting during the Palaeolithic.

## Additional Information

**How to cite this article**: Kübler, S. *et al.* Animal movements in the Kenya Rift and evidence for the earliest ambush hunting by hominins. *Sci. Rep.* **5**, 14011; doi: 10.1038/srep14011 (2015).

## Supplementary Material

Supplementary Information

## Figures and Tables

**Figure 1:**
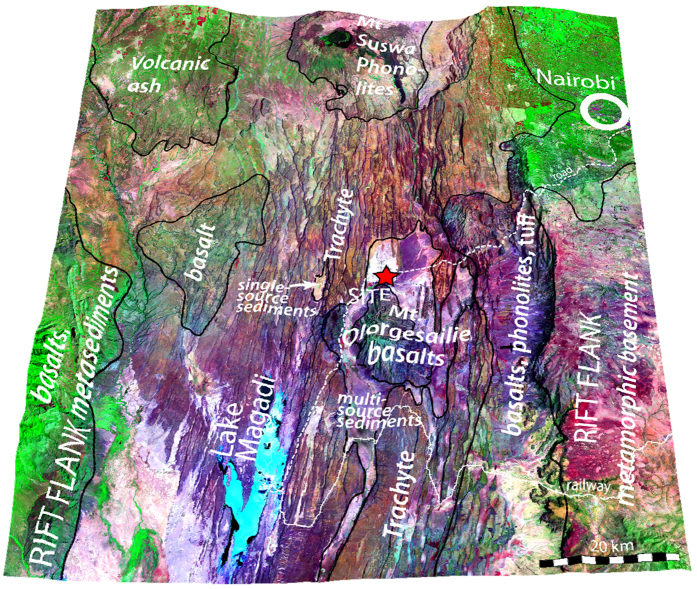
Oblique 3D view of the South Kenya Rift centered on the Olorgesailie hominin site, showing faults and other geological features. The region is heterogeneous with some areas well-vegetated and others with thin vegetation. Thin vegetation can result from soils that do not favour plant growth, but can also result from heavy grazing and browsing of favoured vegetation. The image is based on ETM+ legacy data. Topography from SRTM v4.1 data with a vertical exaggeration of ×8. Geological units based on^16^. A red star indicates the Olorgesailie site. Maps are created by SK and GK using Adobe Illustrator CS 5.1, MaPublisher 9.4.0, Global Mapper 16, and ENVI 5.1.

**Figure 2:**
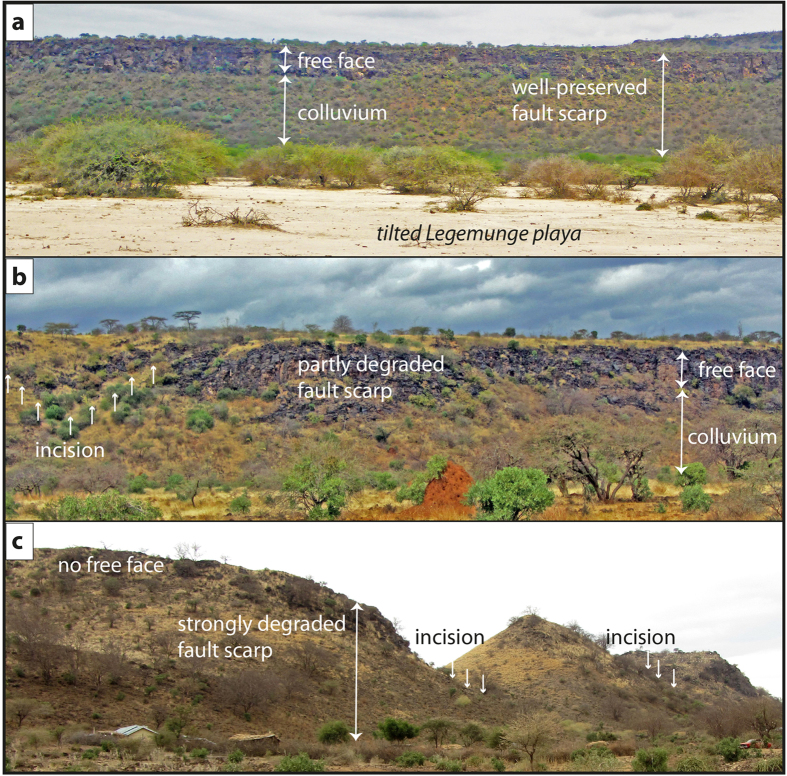
Fault scarps in trachyte of the Olorgesailie region displaying different stages of degradation. (**a**) Well-preserved fault scarp of the normal fault bounding the Legemunge playa to the east, showing the nature of the barrier it would present to movement of large animals. This fault causes the eastward tilt of the playa sediments that contain artifacts and fossils and therefore post-dates their deposition. (**b**) Partly degraded fault scarp southeast of Mt. Olorgesailie subject to first incision and erosion and thus, presumably older than the fault shown in (**a**). Nevertheless, the fault still acts as barrier to large animal movement. (**c**) Strongly degraded fault scarp southeast of Mt. Olorgesailie. The intensive erosion affecting the same rock type implies that this scarp is significantly older than the ones shown in (**a**) and (**b**), respectively. Photographs by authors.

**Figure 3:**
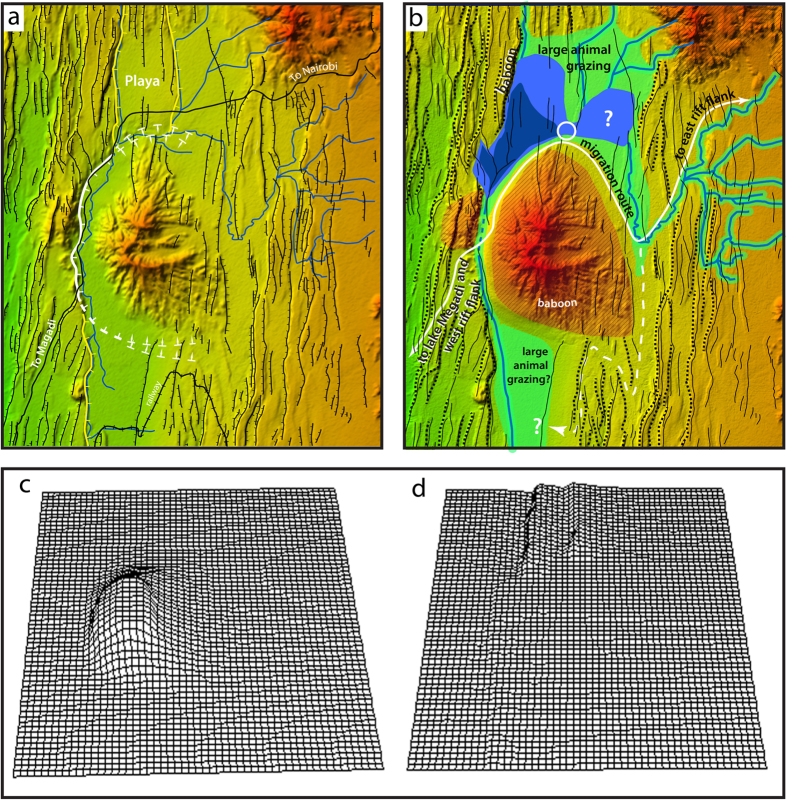
Digital elevation models (DEMs) of Mt. Olorgesailie and the surrounding region. (**a**) Present day topography. Prominent fault scarps are indicated in black and those indicated in yellow and white are used in the modelling of earlier topography. The yellow faults are young faults as indicated by the light colour of the desert varnish, and post-date the lake and the period of hominin activity at Olorgesailie. The white faults result from caldera collapse. Limited access is provided by road and railway to Magadi (indicated in black). (**b**) PalaeoDEM. The white circle indications the position of the Olorgesailie site. The volcanic edifice of Mt. Olorgesailie was already in place and impeded drainage to the south, resulting in the formation of the lake. A possible late lower lake level is indicated in dark blue. Some faults in the trachytes do not cut basalts of the Olorgesailie edifice and therefore clearly pre-date it. The dotted lines show faults that formed barriers to animal movement and are thought to have existed when the site was used by hominins. The drainage system was limited by a barrier in the same place as hypothesized by Behrensmeyer and colleagues^4^, so the barrier could have been higher than the lake without fully blocking drainage from the lake. Likely grazing areas are indicated. Routes to the flanks of the rift negotiable by large animals are shown. Fault scarps and the volcanic edifice would only be accessible to smaller and more agile animals. Maps are created by SK and GK using Adobe Illustrator CS 5.1, MaPublisher 9.4.0, Global Mapper 16, MatLab and ENVI 5.1. Mathematical techniques are described in the Supplementary Material. **(c)** Correction field for caldera collapse. Only the largest effects are visible. (**d**) Correction field for faulting. Only the largest effects are visible.

**Figure 4:**
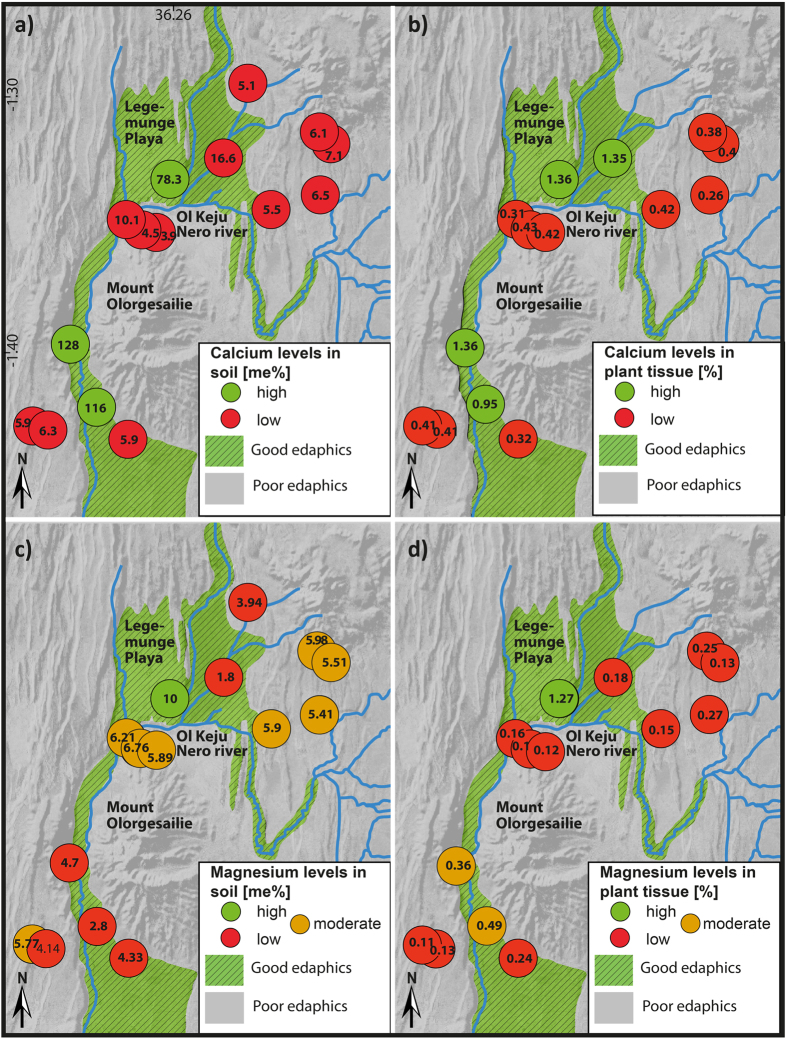
Nutrient levels of soil and plant samples at Olorgesailie. Green shading indicates regions of good edaphics, which occur where there are sediments of composite lithological origin as shown by interviewing local shepherds and soil analyses. (**a**) Calcium levels determined for soil samples; (**b**) Calcium levels determined for plant tissue; (**c**) Magnesium levels determined for soil samples; (**d**) Magnesium levels determined for plant tissue. Maps are created by SK and GK using Adobe Illustrator CS 5.1, MaPublisher 9.4.0 and Global Mapper 16.
