# Supplementary material for: Animal movements in the Kenya Rift and evidence for the earliest ambush hunting by hominins
Source: Sci Rep. 2015 Sep 15;5:14011. doi: 10.1038/srep14011 (PMC4570197; doi:10.1038/srep14011)
Supplement: Supplementary Information [file srep14011-s1.pdf]

Supplementary Material:

Animal movements in the Kenya Rift and evidence for the earliest ambush hunting by hominins

Simon Kübler[1], Peter Owenga[2], Sally C. Reynolds[3], Stephen M. Rucina[4], Geoffrey C. P. King[5]*

Affiliations:

1. *Department of Earth and Environmental Sciences, Ludwig-Maximilians-University Munich, Germany.*
2. *Kenyan National Agricultural Research Laboratory (KALRO-Kabete) Nairobi, Kenya.*
3. *Faculty of Science and Technology, Bournemouth University, Bournemouth, UK, BH12 5BB*
4. *Department of Earth Sciences, National Museums of Kenya, Nairobi, Kenya.*
5. *Labo Tectonique, Institute de Physique du Globe de Paris, Paris, France.*

Corresponding author. Email: king@ipgp.fr.

Overview of the Supplementary Information

1.0 Geology of the study area - text file with additional illustration

2.0 Reconstructing the palaeolandscape - text file with additional illustrations and method descriptions

3.0 Edaphics, nutrient deficiency and associated animal movements in Kenya - text file and table

4.0 Soil analysis - text file with additional illustration, table and method descriptions

5.0 Exploitation of prey species at Olorgesailie - text file with additional illustration and table

1.0 Geology of the study area

The Olorgesailie study area in the southern Kenya Rift is dominated by intermediate to basinic volcanic rocks of Tertiary and Quaternary ages (Figure S1). Older rocks only crop out at the southeastern corner of the study area comprising Precambrian granitic orthogneiss and quartzite of the Mosambique Belt. Apart from the Olorgesailie volcano that consist of Plio-Miocene basalt, trachyte and nephilinite, and basalt and phonolite at the eastern rift flank the area is widely covered with Pleistocene flood trachyte[1] (Magadi Trachyte, Table S1). Complex faulting of the flood trachyte resulted in a complex

topography of numerous fault blocks comprising horsts and grabens that are very little modified by erosion[2]. Lakebeds are found on the Legemunge Playa north of Mount Olorgesailie and around Lake Magadi. In contrast to Lake Magadi, no active sedimentation takes place on the Legemunge playa today. Here, Pleistocene lakebeds crop out several meters above the active riverbed of Ol Keju Nero River that incises into the Legemunge Playa and underlying basalt directly north of Mount Olorgesailie (Figure S1).

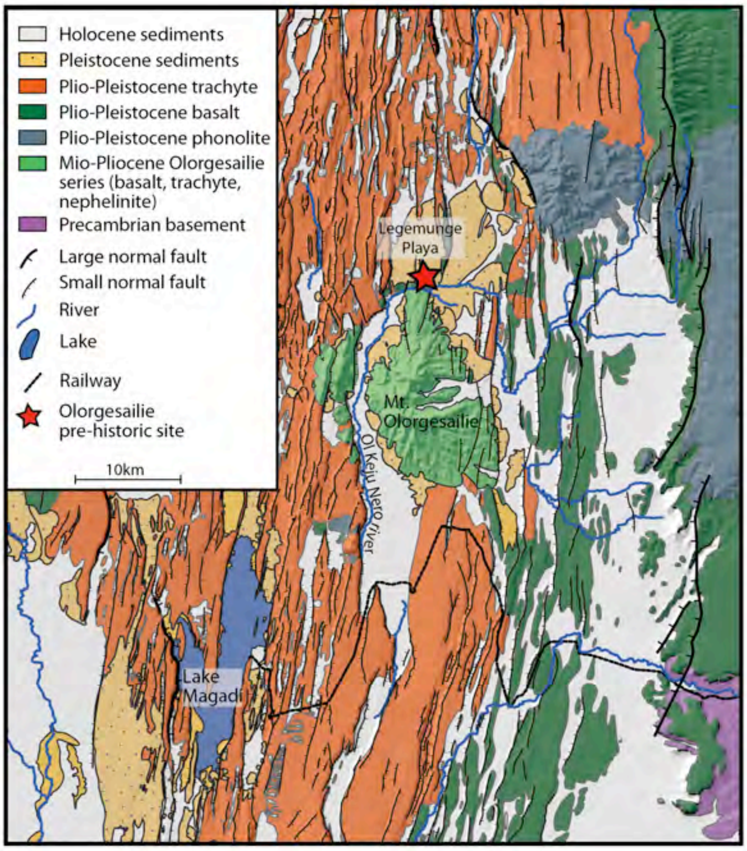

Figure S1. Simplified geological map of the Olorgesailie region comprising the main lithological units; modified from the geological map of the southern Kenya Rift[1]. Additional Geomorphological and sedimentary features have been added from mapping on satellite images. For age constraints on the geological units (see table S1). Prepared using Adobe Illustrator CS5 (15.1.0) and MaPublisher 9.4

Table S1. Age constraints on the main geological units of the Olorgesailie region.

**Age**	**Lithology**	**Unit**	**Dating**
0-? ka	River sediments	Ol Keju Nero River sediments	
9-96 ka	Playa	High Magadi Beds and Chert Series	U/Th[3]
0.2-1.2 Ma	Playa	Olorgesailie Lake Beds, Legemunge Playa	Ar/Ar[4]
0.7-1.4 Ma	Flood Trachyte	Magadi Trachyte	Magnetostratigraphy and K/Ar[5]
1.4-1.79 Ma	Basalt	Ol Tepesi Basalt; Ol Keju Nero Basalt	Magnetostratigraphy[2,5]
2.2-2.7 Ma	Basalt, Nephelinite, Trachyte	Olorgesailie Series	K/Ar[2]
> 3Ma	Basalts, Trachytes	Turoka, Ngong, Kirikiti, Sonjo, Sambu, Naitiami	[6]

2.0 Reconstructing the paleolandscape

Core to our analysis is a reconstruction of the digital elevation (palaeoDEM) at the time of hominin occupation between 1.2 and 0.5 Ma. This is possible in this case because the main components of landscape variability are tectonically and volcanically defined.

The starting point for reconstructing an earlier landscape is a present day Digital Elevation Model (DEM) in this case derived from 90m Shuttle Radar Topographic Mission (SRTM) data. This is then corrected by adding or subtracting a correction displacement field (CDF) across the DEM to restore earlier elevations for each cell within this model, modelled here at a sub-km resolution. To create CDF requires information on: (1) fault motion that has caused uplift, subsidence and flexure; (2) erosion and deposition of sediment modifying the land surface; (3) extrusion of magma to produce lava flows or volcanic edifices; and (4) subsurface movement of magma that may have resulted in uplift and/or subsidence. This information is available with varying degrees of accuracy across the study area. Each of these components needs to have a temporal dimension to allow the CDF to be determined for a specific time interval. This need not however be absolute age determinations since the relative age constrainst are in most cases, as here, sufficient. What is essential in attempting such a reconstruction successful is that the CDF and temporal constraints needs to be sufficiently accurate to provide a paleolandscape reconstruction of value and in this case for a period of known occupation (1.2-0.5 Ma). The dating control for our reconstruction is provided by the Legemunge Playa lake beds which contain the record of hominin occupation and record the presence of the palaeolake. We recognise four key elments:

1 Tilting of the Legemunge playa lake beds. This has resulted from motion on two faults indicated in orange in Figure S2a. This motion post-dates the deposition of the lake beds and hence hominin exploitation of the area under consideration. The mechanism by which they were tilted is shown schematically in Figure 2 b and c and discussed in greater detail below.

2 The lake, which resulted in Legemunge playa was dammed by the Mount Olorgesailie volcanic edifice. The oldest dates for the Olorgesailie beds indicate the first formation of the lake and are consistent with some dating of the Olorgesailie basalts (Table S1).

3 The draining of the lake resulted from faulting (Figure S2, shown in white and in cross section in d) that down dropped much of the Olorgesailie volcanic edifice. Commencement of this down faulting is not known, but the draining of the lake determines when fault motion was sufficient to allow water to drain to the south and it is therefore constrained by the upper most data for the lake beds (Table S1).

4 Trachyte lava flows underlie the volcanic edifice and are extensively cut by N-S faults. The exact dating of the extensive faulting is not known. Some of the faults do not cut the overlying basalts of while others do. As noted above and the maximum age of the faults tilting the playa is dated by the youngest Olorgesailie beds. Continued seismic activity indicates that faulting continues today. It is reasonable to consider that many fault scarps in the region existed at the same time as the lake. The

relative ages of faults can be judged by: (A) the degree of erosion both observed in the field and on satellite images- provides a qualitative measure of relative ages, such that older fault scarps should show greater degradation; (B) the known ages and level of erosion of the faults that have tilted the Legemunge playa; and (C) the degree of desert varnish coating on fault surfaces observed in the field.

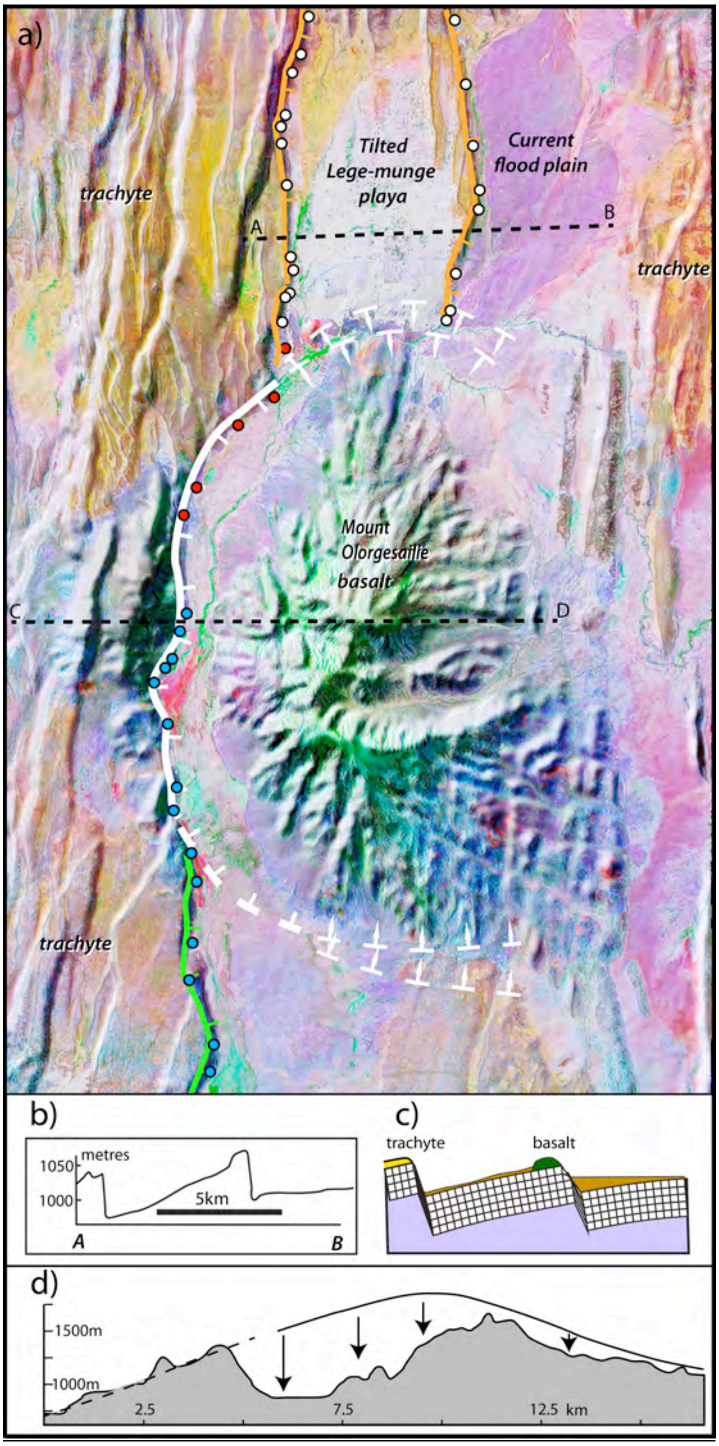

Figure S2. Modified ETM+ Landsat legacy image.

Faults that are back-slipped to reconstruct earlier topography are shown and Small circles indicate segment ends in the modelling. The contours are used to correct for caldera collapse. Inset (B) shows a topographic section A-B and (C) shows a 2D numerical model of the faulting. Inset shows a topographic section C-D and an estimated profile prior to collapse. Further detail of the restoration process is described below. Prepared using Adobe Illustrator CS5 (15.1.0) and MaPublisher 9.4. Topographic shading from SRTM v4.1 data

2.1 Modelling Critical to the above reconstruction are two modelling stages, one associated with the fault dislocations and a second with the collapse of the volcanic edifice.

King and colleagues[7,8] proposed that geological (tectonic) structures can be understood to be the consequence of repeated earthquakes. This introduced a new way to relate data from earthquakes to geological data and suggested new approaches to fault modeling. This work and a number of precendent publications treated the lower crust as being viscous and the upper (seismogenic) crust as a thin elastic plate with a very small "effective elastic thickness". The effective elastic thicknesses found when modeling data were considered to result from weakening the crust between earthquakes. Using a rich data set from the Gulf of Corinth, Armijo and colleagues[9] used boundary element methods to consider a finite thickness crust overlying a denser fluid lower crust. The model fully included surface unloading and loading resulting from erosion and deposition of sediment. They showed with this model that it was only possible to fit observations if the elastic crust had an extraordinarily (unrealistically) low modulus. On the other hand the data could be fitted with a simple elastic half-space model[9]. Figure S3a and b show respectively a half-space model and a finite thickness plate model. Other authors have explored both approaches but within the community that studies earthquakes the concept of a viscous lower crust is losing favor making a model with a strength retaining lower crust more reasonable[10]. Viscous flow in the mantle does respond to surface loading but at scales of hundreds much greater than the few tens of kilometers at Olorgesailie. We therefore use dislocations in a half-space to model faulting using analytic expressions[11]. Whatever method is used motion on normal faults results in downward flexure of the hanging wall and upward flexure of the footwall (Figure S3a and b). The relative amounts depend on fault dip and the extent of deformation perpendicular to the fault strike depends on the depth to which faulting extends (Figure S3c)

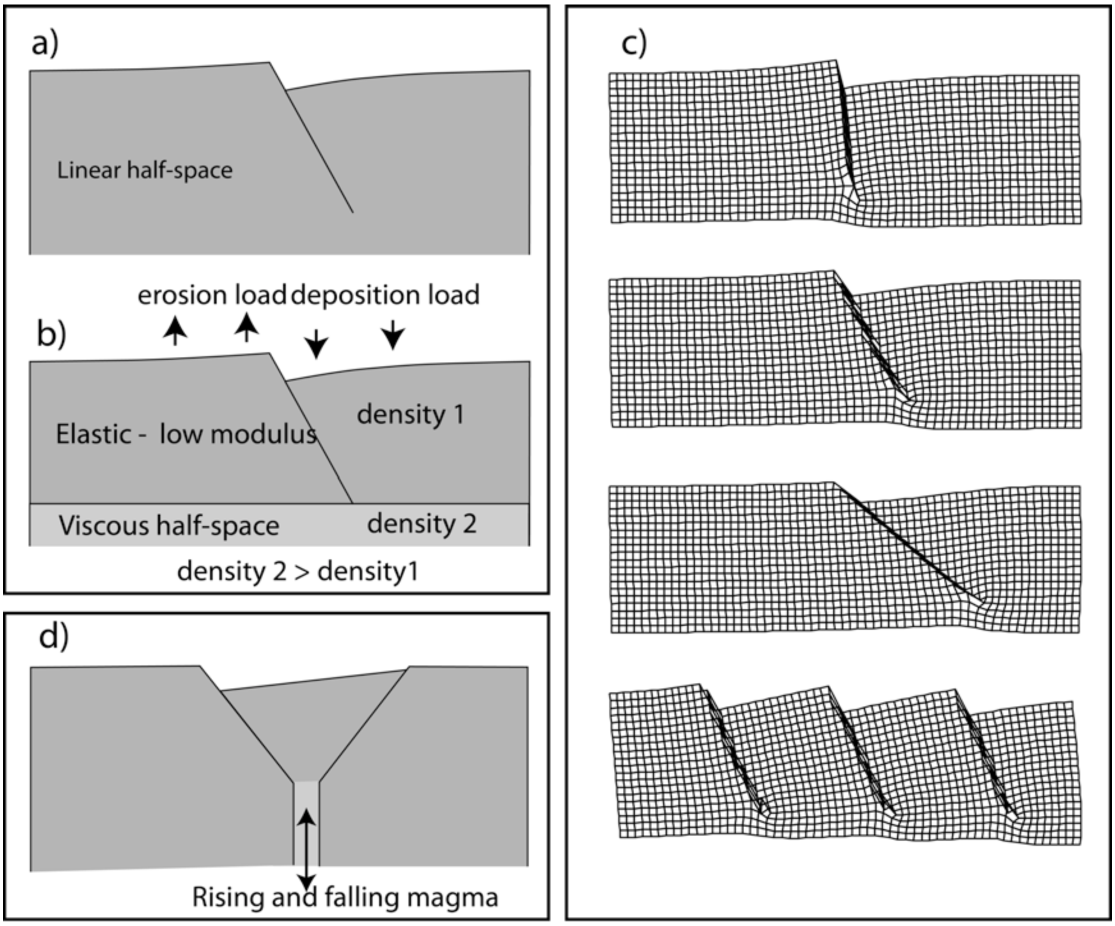

Figure S3. Modeling normal faults. (A) A fault cutting an elastic upper crust overlying a viscous lower crust. (B) A fault in an elastic half-space. (C) The effect of changing fault parameters. Note that fault displacement causes the faults to rotate causing the originally vertical (90°) fault to become slightly off vertical. The rectangular grid has a 0.5km spacing before deformation.
(D) Faulting associated with magma movement.

Magma chambers or dykes in which the magma level can change may result in blocks subsiding (Figure S3c). A characteristic feature of collapse faulting is that it is not associated with the flexure no to the uplift to subsidence ratios described above. Figure S4 shows an example of collapse from the Asal Rift (Djibouti)[12]. Dislocation methods can be adopted by introducing contraction elements at depth to model magma motion and this approach has been adopted here to model high-resolution geodetic data associated with the Olorgesailie volcanic edifice. However, simpler methods can be used to approximate uplift and subsidence at the surface. For the region shown in Figure S4 this has been done by reconstructing the form of the Fieale volcano[13]. For Olorgesailie a displacement field is estimated to correct for the caldera collapse.

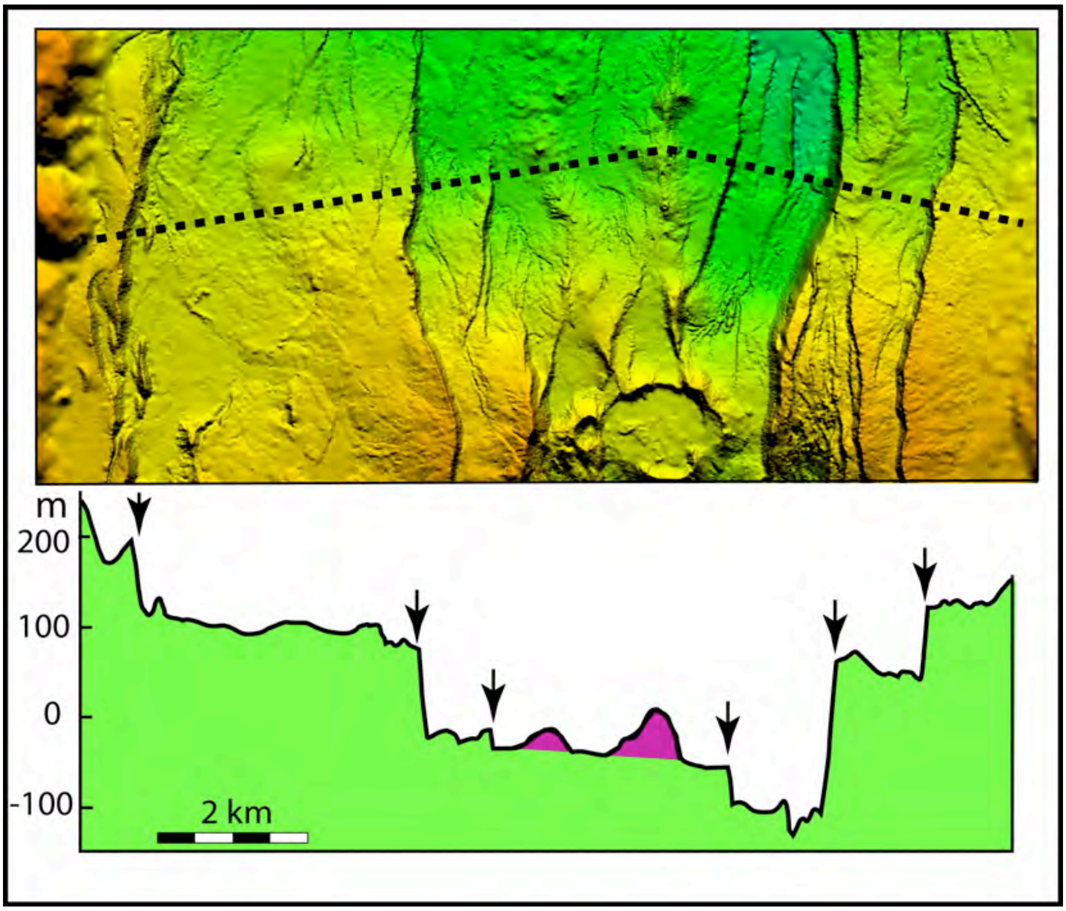

Figure S4. Faulting in the Asal rift, Djibouti. The faults shown cause down-drop without flexure and uplift. The features shown in Mauve are small cones associated with an eruption in 1978. The clarity of the faulting and the form of the Fieale volcanic edifice has allowed the previous landform to be reconstructed[12,13]. Prepared using Adobe Illustrator CS 5.1 and MaPublisher 9.4.0.

The most straightforward approach to modelling the edifice drop is to assume that the west playa fault (yellow in Figure S2) is continuous with the fault cutting the west side of the volcanic edifice (white in Figure S2) and with the same fault extending further to the south (green in Figure S2). It becomes clear from dislocation modeling (an example is shown in Figure S7) that offset values for the white fault will create a landscape that would retain a lake. Removing the footwall uplift would allow water to drain to the west. Furthermore the fault offsets would have to be much greater (>4x) than that on the faults to the north and south to restore the form of the volcanic edifice. The faulting can be understood better if it is associated with a half caldera collapse similar to that observed for Mount Suswa volcano to the north (see: Google Earth-1.117°S, 36.318°E). Previous workers do not appear to have considered this possibility highlighted by modelling. However, the arcuate form of the faulting could have been a clue. This suggests that the northern part of the volcanic edifice and the playa has been subject to down warping as indicated in Figure S2 by white symbols. The caldera collapse can be corrected by creating displacement values to allow for the subsidence. This is done by plotting numerous cross-sections. Examples are shown in Figure S6 and the form of the earlier morphology estimated at a sufficient number of points to allow the creation of a contour

map of the correction required as shown in Figure S8. This is converted to CDF allowing for subsidence of the edifice, erosion and sedimentation. Figure S9 shows the corrected edifice with sections to compare with those shown in Figure S6. The contours and CDF were progressively adjusted such that the final form of the original edifice is reasonable. As noted above, if a region is no larger than a few 10s of km^2, the unloading and loading effect of erosion and deposition of material can be ignored. The DEM can be corrected by finding CDF to correct for material eroded or added by sedimentation. The tilting of the playa can be seen in Figure S2b. The responsible faults are shown in orange in Figure S2a and small circles indicate fault ends (numbered) used for modelling. These segments are used to calculate the faults slips required to create a CDF to restore the playa. A wide range of models with different values for fault strike, dip and slip were tested. In each case a cross section was drawn. An example is shown in Figure S4. For a range of possible values (depths 5 - 10km, dips 60 - 80°) the results are similar. The average altitude varies by less than ± 20m.

1.2 Modelling accuracy.

The reconstruction of the playa to create the conditions when it could hold a lake is straightforward. Only the two faults identified (yellow in Figure S2) could have been responsible for the tilting and since the lake could not have existed with the present day configuration this dates when their motion started. There has been some erosion of the playa but nonetheless its height and form is well constrained by the reconstruction. The correction for the caldera collapse is well constrained only for the west of the edifice; elsewhere values are chosen to be reasonable and does not affect interpretations of how the landscape was used. The most important feature is to identify how the lake was impounded. The correction shown in Figure S7 is sufficient to form a barrier that would impound the lake and identifies where the lake could have extended south of the playa. Identifying caldera collapse as the process that allowed the lake to drain implies that some downwarping occurred between the playa and the northern part of the volcanic edifice. This suggests that the Olorgesailie sediments would have also been warped. The modelling we have carried out is based on a 90m resolution DEM, which is not sufficient to link with the detailed mapping of the sediments. However when 3 meter or better DEM's become available this may be possible if the plane table mapping of the geology[14] was sufficiently accurate.

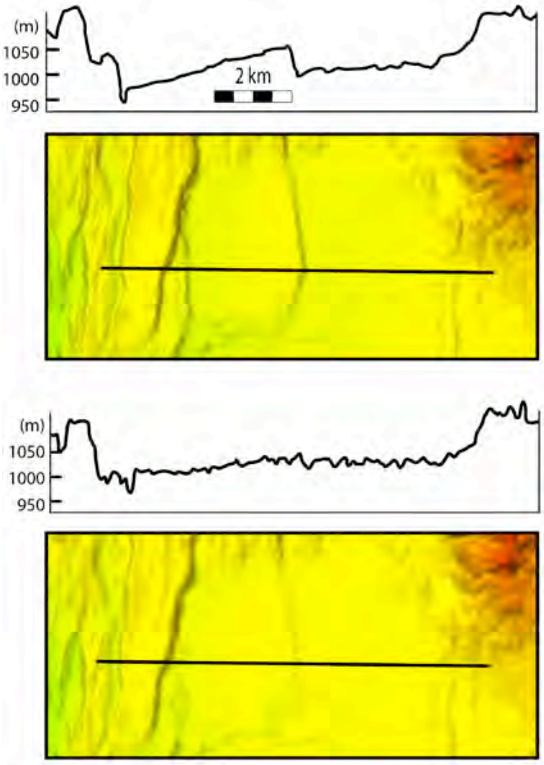

West Fault

Start	Finish	Dip(km)	Depth(km)	Disp(km)
3	4	70.0	7.5	0.06
4	5	70.0	7.5	0.06
5	6	70.0	7.5	0.06
6	7	70.0	7.5	0.06
7	8	70.0	7.5	0.06
8	9	70.0	7.5	0.08
9	10	70.0	7.5	0.08
10	11	70.0	7.5	0.08
11	12	70.0	7.5	0.08
12	13	70.0	7.5	0.08
13	14	70.0	7.5	0.08
14	15	70.0	7.5	0.08
15	16	70.0	7.5	0.08

East Fault

Start	Finish	Dip(km)	Depth(km)	Disp(km)
39	40	70.0	7.5	0.03
40	41	70.0	7.5	0.03
41	42	70.0	7.5	0.04
42	43	70.0	7.5	0.06
43	44	70.0	7.5	0.05
44	45	70.0	7.5	0.04
45	46	70.0	7.5	0.04

Figure S5. Cross sections of the playa before and after correction. The table indicates the fault slips required to remove the playa tilt. Segments are shown and numbered in Figure S2a. Prepared using Adobe Illustrator CS5 (15.1.0) and MaPublisher 9.4. Topographic shading from SRTM v4.1 data.

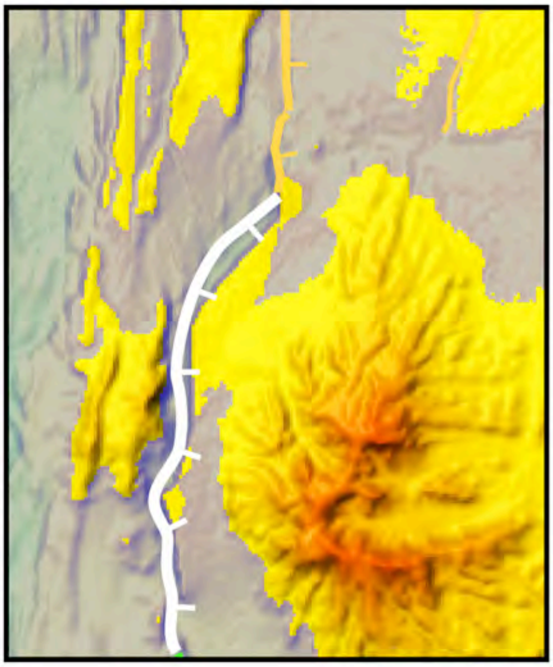

White Fault

Start	Finish	Dip	Depth	Disp (km)
16	17	70.0	7.5	0.24
17	18	70.0	7.5	0.24
18	19	70.0	7.5	0.24
19	20	70.0	7.5	0.24
20	21	70.0	7.5	0.24
21	22	70.0	7.5	0.24
22	23	70.0	7.5	0.24
23	24	70.0	7.5	0.24
24	25	70.0	7.5	0.24
25	26	70.0	7.5	0.24

Figure S6. Dislocation modelling of the faults to the west of the volcanic edifice. The values required to remove the offset of the volcanic cone are ~4 times greater then on the playa fault to the north. Grayed region indicates a lake level that would inundate the playa. However such a lake would be unconstrained to the west or south. The problem arises as a result of removing the footwall uplift associated with faulting. Prepared using Adobe Illustrator CS5 (15.1.0) and MaPublisher 9.4. Topographic shading from SRTM v4.1 data

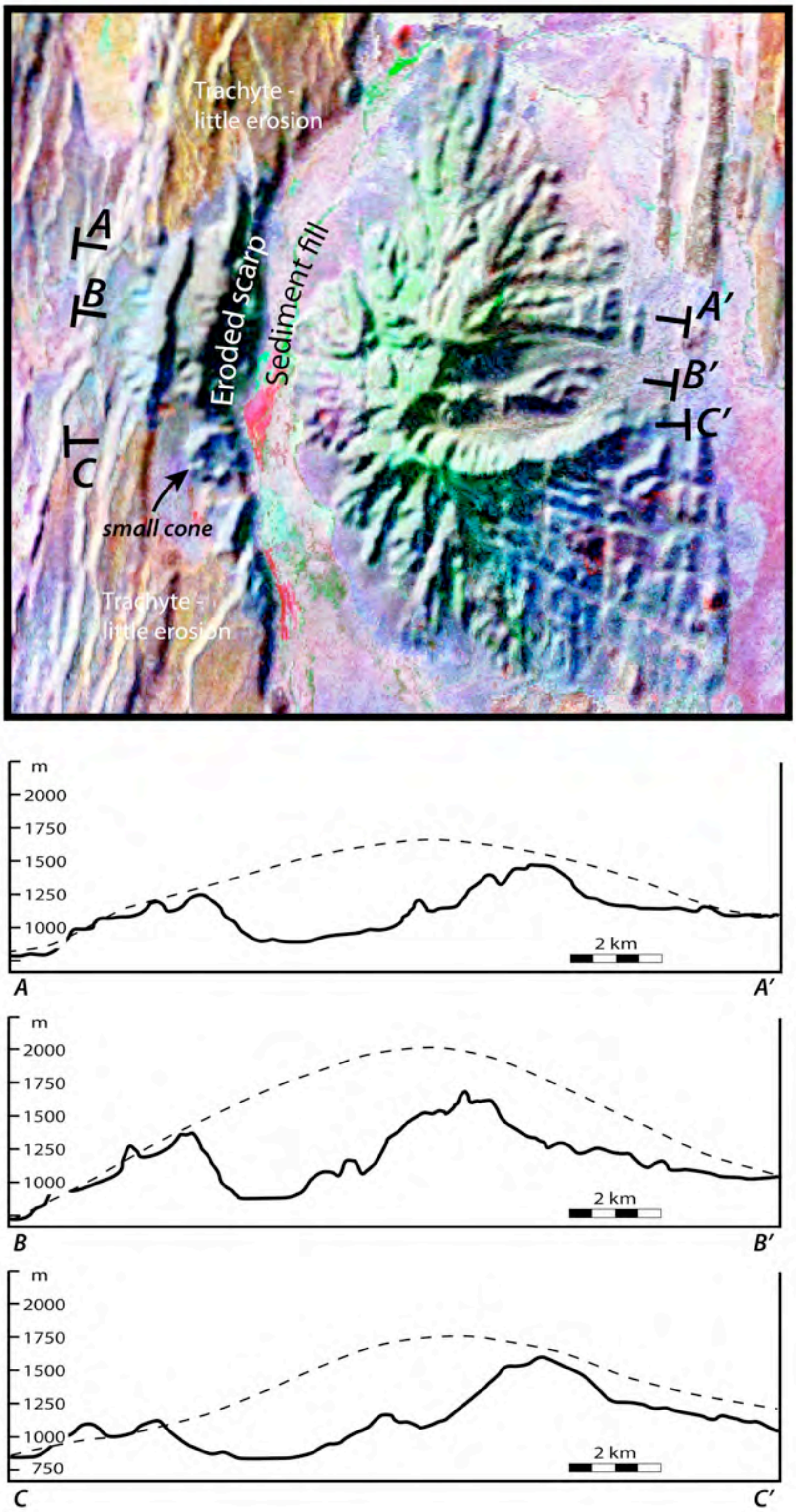

Figure S7. An example of sections across the volcanic edifice. Dashed lines indicate a reasonable (first guess) original profiles. Elevation differences for a series sections are used to create a contour map of the corrections needed to correct for caldera collapse. Prepared using Adobe Illustrator CS5 (15.1.0) and MaPublisher 9.4 and Global Mapper 16. Image from NASA ETM+ imagery. Topographic shading from SRTM v4.1 data

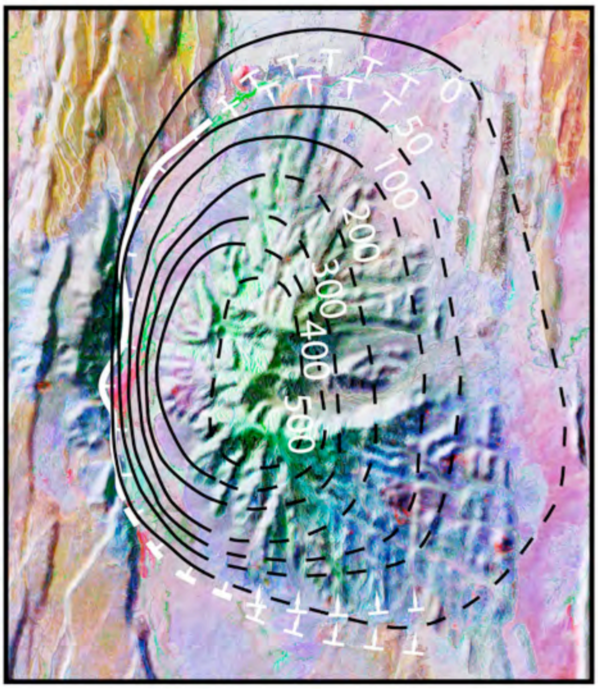

Figure S8. Contours determined from many cross sections (e.g. Figure S5). These are reasonably well constrained to the west and north of the edifice. Elsewhere no information is available. The contours are chosen to produce a reasonable reconstruction. Any reasonable values are consistent with interpretations of the landscape in the main text. Prepared using Adobe Illustrator CS5 (15.1.0) and MaPublisher 9.4 and Global Mapper 16. Image from NASA ETM+ imagery. Topographic shading from SRTM v4.1 data.

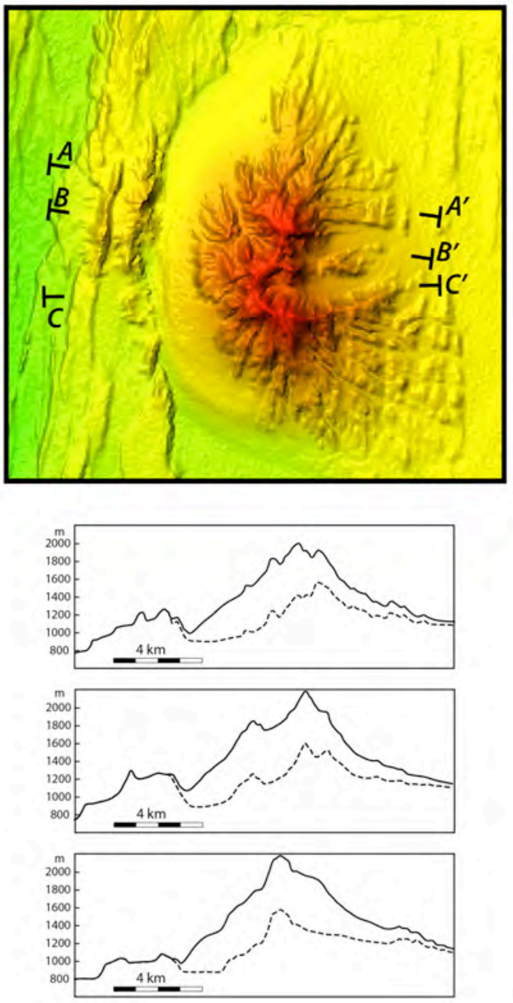

Figure S9. reconstructed topography. The cross-sections follow the same lines as in Figure S6. The dashed lines are sections before correction and solid line are for the palaeoDEM. Prepared using Adobe Illustrator CS5 (15.1.0) and MaPublisher 9.4 and Global Mapper 16. Topographic shading from SRTM v4.1 data.

3.0 Edaphics, nutrient deficiency and associated animal movements in Kenya

The term edaphics describes the quality of a soil regarding its physical and chemical composition and how this supports or limits plant growth as well as the provision of nutrients that is - via plant take-up - essential for the animal diet. The physical properties such as texture, permeability and grain size distribution mainly control the stability and water retentivity of a soil[15,16]. Chemical properties control the availability of macronutrients and trace elements for plant take-up and animal nutrition[15]. The main controlling factors for edaphics are: (1) parent material (bedrock lithology or sediment composition); (2) climate (mainly rainfall and temperature); (3) soil formation processes (leaching, removal/accumulation of clay, gleying, etc.); and (4) relief[15].

Table S2. Macronutrient and trace element deficiency levels for grazing and browsing mammals and associated diseases.

Element	Deficiency levels (plant tissue)	Known deficiencies in Kenya	Associated diseases and health issues[17]	Interactions with other minerals
Ca	**<0.4-0.8%**[17]	Bungoma and Trans Nzonia districts[18]	Rickets, osteoporoses, retarded growth, decreased food consumption, nutritional secondary hyperparathyroidism (associated with excessive *P*)	Excessive *Mn* and *P* form insoluble complexes with *Ca*; the best *Ca:P* ratio for mammal nutrition is 1:1 to 1:2[17]
Mg	**>0.2-0.6%**[17]	low *Mg* levels in some areas[19]	Tetany, convulsion, vasodilation, reduced appetite and weight loss	
P	**<0.2-0.6%**[19]	everywhere except Nakuru/Gilgil[18,19]	Rickets, reduced body growth, reduced productivity, loss of appetite/abnormal appetite, weakness and death	Excessive *Fe*, *Al* and *Mg* form insoluble complexes with *P*[17]
N	**<1.5%**[15]	no		
Na	**<0.2-0.25%**[19]	Very low Na levels throughout Kenya, therefore nutrient supplementation by farmers[19]	Reduced growth, softening of bones, corneal keratinization, gonadal inactivity, loss of appetite, weakness, blood plasma decrease	
K	**<0.5-0.7%**[17]	no	Muscle weakness, cardiac and respiratory weakness, degeneration of kidneys	
Cu	**3-5ppm**[17]	Nakuru, Naivasha, Njoro, Rongai, Solai regions[19,20]	Anemia, Retarded growth, reduced appetite, reduced hair growth, diarrhea, bone deformation, reduced productivity	Absorption of *Cu* is affected by high levels of other ions, mainly *Zn* and *Mo*[16,17]
Mn	**8.5-50ppm**[17]	No	Weight loss or reduced growth, impaired reproduction, weakness, nervous disorder, bone malformations	
Zn	**10-70ppm**[17]		Retarded growth, hair loss, weight loss, reduced reproduction, impaired wound healing	High *Ca* levels can reduce *Zn* availability[16,17]
Fe	**35-50ppm**[16]		Anemia, listlessness, weight loss	see *Ca*

Required nutrient levels in fodder depend on mammal species, age, sex, maturity as well as behavioural stress and/or reproductive condition[17]. Animal diseases related to nutrient deficiencies (Table S2) occur mostly if animals are exposed to deficient fodder over long periods of time. To avoid this, grazing and browsing animals in the Rift Valley migrate throughout the year from one nutrient source to the next depending on the seasonal availability and their nutritional needs at a given time. A study in the Serengeti, Tanzania[21] showed that a major driving force for the great wildebeest migration is a constant level of slight undernutrition, particularly related to soluble Phosphate, Calcium and Magnesium. Nutrient deficiency and associated animal diseases are fairly common in the East African Rift[19,20] and if one region is rich in one mineral source it often lacks

another. Soils in the Lake Nakuru region[20] in the central Rift Valley, for example, are characterised by severe copper deficiency leading to diseases in both wildlife and livestock (Table S2). On the other hand, this region is fairly rich in Sodium and soluble Phosphate - nutrients that are deficient in most other areas in the Kenya Rift[22]. This means for most regions in the Kenya Rift, animals have to move in the course of the year, because if they stayed in one place for too long, health problems related to under nutrition would be the result.

4.0 Soil analysis

The objective of soil analysis at Olorgesailie was to get information on soil edaphics in relationship with the individual rock and sedimentary units. Soil sampling sites (Figure S10) were carefully chosen based on field observations and information from geological maps[1]. Sampling was carried out by Peter Owenga (Kenyan Agricultural Research Institute). Soil fertility samples were taken from the uppermost soil horizon (<25cm). Soil and plant tissue analysis was carried out by the Kenyan National Agricultural Research Laboratory (KARI-Kabete). Results are shown in Tables S3 and S4, respectively. Before analysis the samples were air dried and sieved through a 2-mm sieve. Soil pH was measured in 1:2.5 soils to water mixture, using the relevant electrodes[23]. Organic carbon was oxidized with concentrated H_2SO_4 and K_2CrO_7 and determined calorimetrically[24]. Total N was determined using the method provided by Okalebo and colleagues[25]; cation exchange capacity (CEC) and exchangeable cations were extracted using 1N ammonium acetate at pH 7.0, followed by flame photometry for the determination Na, K, Mg and Ca, using a2 flow analyser[25]. Soil texture was determined using the hydrometer method[23].

A total of 15 soil samples (Table S3) and 12 plant tissue samples (Table S4) were tested for concentration of the following trace elements and nutrients: calcium *Ca*, copper *Cu*, iron *Fe*, manganese *Mn*, magnesium *Mg*, nitrogen *N,* potassium *K* (soil samples only, Table S3), sodium *Na* (soil samples only, Table S3), phosphorus *P*, and zinc *Zn* (Table S1, S2). Further, soil samples were tested for pH-value, electric conductivity, and total organic carbon (Table S3). Sample locations are shown in Figure S10.

To test whether nutrient concentrations of soils developed on similar rock units elsewhere in the Kenya Rift match our observations in Olorgesailie, we sampled soils on trachytic and basaltic bedrock in the Lake Baringo region (Table S5). The results of plant tissue analysis (Table S4) support our hypothesis that trachyte soils generally lack calcium and magnesium whereas particularly soils on lake sediments provide reliable sources of such nutrients.

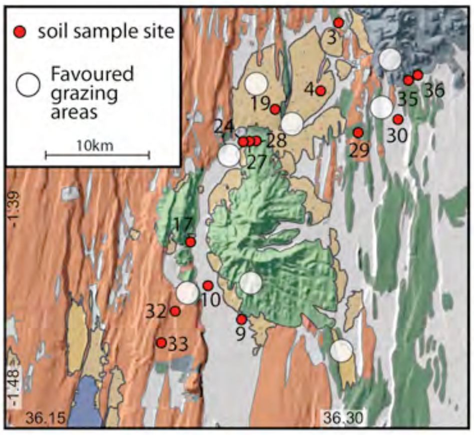

Figure S10. Location of sites for soil sampling (red dots) and grazing areas (white circles) favoured by local sheeps and goats. The white circles represent an area approximation based on information gained from interviewing heads of Masaai pastoral families. The map key of the geology is explained in Figure S1 The map is an extract of main text Figure 1.

Table S3. Nutrient and trace element levels for soil sample analysis in the Olorgesailie region. Al: Alluvium; Pl: playa deposits; Vb: basalt; Vp: phonolite; Vt: trachyte; me%: milliequivalents per 100g soil.

Waypoint	Lithology	Ca me%	Cu ppm	K me%	Fe ppm	Mg me%	Mn me%	Na me%	N %	P ppm	Zn ppm	C org %	pH	el. condmS/cm
3	Vt	5,1	1,72	0,9	24,8	3,94	0,31	0,24	0,1	3	1,8	0,8	7,97	0,25
4	Al	16,6		1,4		1,8		0,1				0,9	7	0,06
9	Al	6,5	3,13	2,47	14,5	5,41	0,15	0,6	0,1	13	2,71	0,9	7,4	0,35
10	Al	116		2,9		2,8		0,4				1,6	7,7	0,39
17	Al	128		4,3		4,7		0,9				1,5	8	0,25
19	Pl	78,3		4,7		10		0,3				1,1	8,5	0,15
24	Vb	10,1	1,4	2,39	16,4	6,21	0,12	0,78	0,1	2	2,5	0,9	8,02	0,54
27	Vb	4,5	2,58	0,86	21,2	6,76	0,16	0,32	0,1	1	3,64	1,27	7,86	0,32
28	Vb	3,9	5,26	0,78	58	5,89	0,11	0,26	0,1	4	23,1	1,16	8,62	0,19
29	Vb	5,5	5,03	1,62	54,9	5,9	0,19	0,32	0,1	90	32,6	1,2	6,82	
30	Al	5,9	3,06	1,4	45,8	4,33	0,21	0,42	0,1	145	28,6	0,76	6,97	
32	Vt	6,3	1,22	1,84	17,7	4,14	0,22	0,4	0,1	5	3,35	0,76	7,59	0,29
33	Vt	5,9	8,67	1,48	99,3	5,77	0,17	0,44	0,1	75	5,17	1,05	6,99	
35	Vp	6,1	2,64	1,7	15,2	5,98	0,22	0,4	0,2	2	1,96	1,95	7,45	0,26
36	Vb	7,1	1,28	1,4	27,3	5,51	0,21	0,6	0,2	2	1,63	2,16	7,33	0,32

Table S4. Nutrient and trace element levels for plant tissue analysis in the Olorgesailie region.

Waypoint	Lithology	Ca %	Cu ppm	Fe ppm	Mn ppm	Ma %	N %	P %	Zn ppm
4	Al	1,35		495	202	0,18	1,75	0,41	8
9	Al	0,26	47,3	4507	153	0,27	1,05	0,24	47,3
19	Pl	1,36		281	303	1,27	1,75	0,28	8,67
24	Vb	0,31	29,8	644	65,8	0,16	0,7	0,19	29,8
27	Vb	0,43	37,3	1057	37,5	0,1	0,7	0,15	37,3
28	Vb	0,42	22,8	1175	52,8	0,12	0,7	0,08	22,8
29	Vt	0,36	31,2	1905	81	0,15	1,05	0,15	31,2
30	Al	0,32	14,8	1970	62	0,24	1,05	0,25	12,3
32	Vt	0,44	16,2	4335	118	0,13	1,05	0,16	32,7
33	Vt	0,41	12,8	1400	49,5	0,11	1,05	0,11	22,3
35	Vp	0,38	13,8	2970	115	0,25	1,05	0,17	38,7
36	Vb	0,4	14,2	3045	124	0,13	0,7	0,07	37,5

Table S5. Nutrient levels for plant tissue analysis in the Lake Baringo region.

**Location**	**Lithology**	**Ca**	**Mg**	**P**
Lake Baringo West	Trachyte (Tertiary)	0,45%	0.27%	0.32%
Lake Baringo North	Trachyte (Pleistocene)	0.56%	0.32%	0.43%
Lake Baringo West	Basalts (Tertiary)	0.9%	0.76%	0.45%
Lake Baringo East (Chesawenja)	Basanites (Tertiary)	3.26%	0.63%	0.36%
Lake Baringo South	Lakebeds (Pleisto- /Holocene)	3,19%	0,8%	0,54%

5.0 Exploitation of prey species at Olorgesailie

The prey species and predators are based on the species list and abundances, with associated palaeoenvironmental indications (Figure S11) for Olorgesailie Upper Member 1[28].

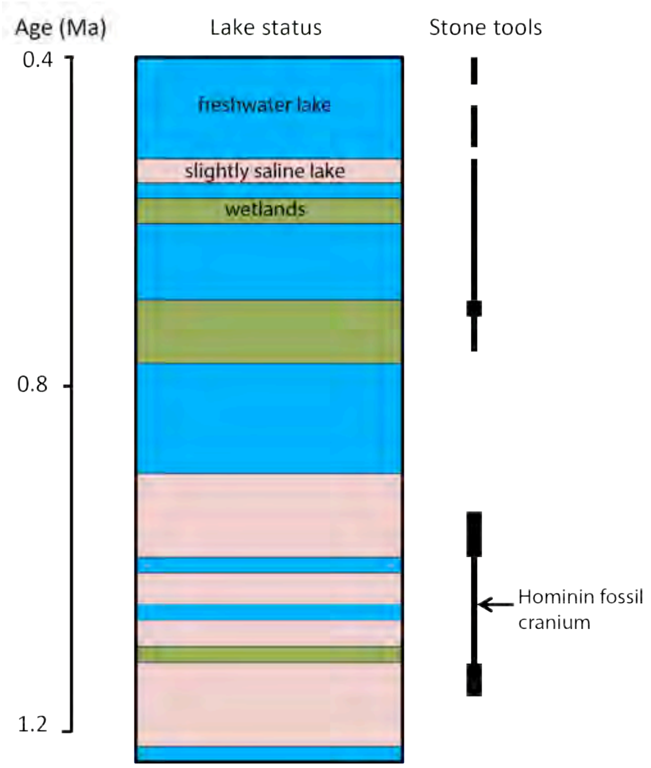

Figure S11. Lake status and distribution of stone tools and artefacts of the palaeoloake north of Olorgesailie. Thickness of black bars is subjectively proportional to frequency of tools in the sediment (modified from[26], age of fossil cranium from[27]). Prepared using Adobe Illustrator CS5 (15.1.0)

Table S6. Palaeofauna found at Olorgesailie site

Family	NISP/MNI* (%)	Species	Locality[29]	Diet and Ecology
Equidae	46-50 / 29- 38	*Equus olduwayensis* **	HH; 15; I3	Grazer, grassland
		Equus grevyi	HH; 15; I3	Grazer, dry grassland, obligate drinker
		Hipparion sp. **	HH; 15; I3	Grazer, grassland
Rhinocerotidae	3-15 / 6-7	*Ceratotherium simum*	HH; 15; I3	Grazer, grassland, obligate drinker
Bovidae	16-29 / 21-23	*Megalotragus* sp. **	HH; 15: I3	Grazer, grassland,
		Connochaetes sp.	HH; 15; I3	Grazer, grassland, obligate drinker
		Alcelaphus sp.	HH; 15; I3	Grazer, grassland,
		Pelorovis sp. **	HH; 15; I3	Grazer, grassland
		Redunca sp.	I3	Grazer, grassland, close to water
		Taurotragus oryx	HH; 15; I3	Grazer and browser, wide range of habitats
		Tragelaphini, medium	HH; 15; I3	? probable browser, riverine forest, (such as *Tragelaphus scriptus*)
		Hippotragini, medium	HH ;15; I3	? Unknown, possibly mixed feeder
Giraffidae	1.1. / 0 -1	*Giraffa* sp.	I3	Browser, savannah woodland
Suidae	3-7 / 4-7	*Metridiochoerus* cf. *andrewsi* **	HH; 15; I3	Grazer, grassland
		Phacochoerus sp.	HH; 15; I3	Grazer, savannah
Hippopotamidae	7-25 / 8-14	*Hippopotamus gorgops* **	HH; 15; I3	Grazer, grassland,
		Hippopotamus amphibius	HH; 15; I3	Grazer, grassland, deep water
Cercopithecidae	1-6 / 4-7	*Theropithecus oswaldi*	HH; 15; I3	Grazer, grassland
		Cercopithecus sp.	HH	Omnivore, wooded habitats
Hyaenidae	0-5 / 0-12	*Crocuta crocuta*	HH	Carnivorous, wide range of habitats,
Viverridae	1.1. / 0-7	*Mungos* sp.	HH	Insectivorous, wooded savannah
		Herpestes ichneumon	15	Carnivorous, dense vegetation around water
Elephantidae	0-99 / 0-7	*Elephas recki* **	HH; 15; I3	Grazer, grassland

(*) NISP: number of identified specimens; MNI: minimum number of individuals
(**) denotes extinct species; Faunal data[29]: HH = Hyena Hill; 15 = Site 15; and I3 = Locality I3. Ecological data from extant species[30] Palaeoecological data derives from isotopic studies of fossil enamel of the species concerned from various African localities[31-36].

References

1 Guth, A. & Wood, J. Geological Maps of the southern Kenya rift (Nairobi, Suswa, Magadi). *Michigan Technical University. Digital map and chart series DMCH016, USGS, Boulder, Colorado* (2013).
2 Baker, B. H. & Mitchell, J. G. Volcanic stratigraphy and geochronology of the Kedong–Olorgesailie area and the evolution of the South Kenya rift valley. *J. Geol. Soc. London* **132**, 467-484 (1976).
3 Behr, H.-J. & Röhricht, C. Record of seismotectonic events in siliceous cyanobacterial sediments (Magadi cherts), Lake Magadi, Kenya. *Int. J. Earth Sci.* **89**, 268-283 (2000).
4 Deino, A. & Potts, R. Single-crystal 40Ar/39Ar dating of the Olorgesailie Formation, Southern Kenya Rift. *J. Geophys. Res.-Sol. Ea. (1978–2012)* **95**, 8453-8470 (1990).
5 Baker, B., Williams, L., Miller, J. & Fitch, F. Sequence and geochronology of the Kenya rift volcanics. *Tectonophysics* **11**, 191-215 (1971).
6 Crossley, R. The Cenozoic stratigraphy and structure of the western part of the rift valley in southern Kenya. *J. Geol. Soc. London* **136**, 393-405 (1979).
7 King, G. C., Stein, R. S. & Rundle, J. B. The growth of geological structures by repeated earthquakes 1. Conceptual framework. *J. Geophys. Res.-Sol. Ea.* **93**, 13307-13318 (1988).
8 Stein, R. S., King, G. C. & Rundle, J. B. The growth of geological structures by repeated earthquakes 2. Field examples of continental dip-slip faults. *J. Geophys. Res.-Sol. Ea.* **93**, 13319-13331 (1988).
9 Armijo, R., Meyer, B., King, G., Rigo, A. & Papanastassiou, D. Quaternary evolution of the Corinth Rift and its implications for the Late Cenozoic evolution of the Aegean. *Geophys. J. Int.* **126**, 11-53 (1996).
10 King, G. C. & Wesnousky, S. G. Scaling of fault parameters for continental strike-slip earthquakes. *B. Seismol. Soc. Am* **97**, 1833-1840 (2007).
11 Okada, Y. Internal deformation due to shear and tensile faults in a half-space. *B. Seismol. Soc. Am.* **82**, 1018-1040 (1992).
12 Stein, R. S., Briole, P., Ruegg, J. C., Tapponnier, P. & Gasse, F. Contemporary, Holocene, and Quaternary deformation of the Asal Rift, Djibouti: Implications for the mechanics of slow spreading ridges. *J. Geophys. Res.-Sol. Ea.* **96**, 21789-21806 (1991).
13 De Chabalier, J.-B. & Avouac, J.-P. Kinematics of the Asal Rift (Djibouti) determined from the deformation of Fieale Volcano. *Science*, 1677-1677 (1994).
14 Shackleton, R. M. Geological map of the Olorgesailie area, Kenya. *J. Geol. Soc. London Spec. Publ.* **6**, 171-172 (1978).
15 Blume, H.-P. et al. *Lehrbuch der Bodenkunde*. (Springer, 2010).
16 Foth, H. D. *Fundamentals of soil science*. (John Wiley and Sons, Inc., 1991).
17 Robbins, C. *Wildlife feeding and nutrition*. (Elsevier, 1983).
18 Jumba, I. O. Tropical soil-plant interactions in relation to mineral imbalances in grazing livestock, *Dissertation Univ. Nairobi* (1989).
19 Howard, D. A. Notes on animal diseases XXIII - Mineral deficiency diseases *E. Afr. agr. forestry j.* **April**, 191-195 (1963).

20 Maskall, J. & Thornton, I. The distribution of trace and major elements in Kenyan soil profiles and implications for wildlife nutrition. *J. Geol. Soc. London Spec. Publ.* **113**, 47-62 (1996).

21 McNaughton, S. J. Mineral nutrition and seasonal movements of African migratory ungulates. *Nature* **345**, 613-615 (1990).

22 Abate, A. Effect of pasture mineral levels on extensive cattle production in Kenya. *African Forage Plant Genetic Resources, Evalulation of Forage Germplasm and Extensive Livestock Production Systems*, 449 (1988).

23 Hinga, G., Muchena, F. N. & Njihia, C. M. *Physical and chemical methods of soil analysis.* (Republic of Kenya, Ministry of Agriculture, National Agricultural Laboratories, 1980).

24 Anderson, J. & Ingram, J. *Tropical Soil Biology and Fertility: A Handbook of Methods*. (CAB International, Wallingford, 1993).

25 Okalebo, J. R., Gathua, K. W. & Woomer, P. L. *Laboratory methods of soil and plant analysis: a working manual.* (Tropical Soil Biology and Fertility Programme Nairobi, Kenya, 1993).

26 Owen, R. B., Potts, R., Behrensmeyer, A. K. & Ditchfield, P. Diatomaceous sediments and environmental change in the Pleistocene Olorgesailie Formation, southern Kenya Rift Valley. *Palaeogeogr. Palaeocl.* **269**, 17-37 (2008).

27 Potts, R., Behrensmeyer, A. K., Deino, A., Ditchfield, P. & Clark, J. Small Mid-Pleistocene hominin associated with East African Acheulean Technology. *Science* **305**, 75-78 (2004).

28 Potts, R. Variables versus models of early Pleistocene hominid land use. *J. Hum. Evol.* **27**, 7-24 (1994).

29 Potts, R. Hominin evolution in settings of strong environmental variability. *Quat. Sci. Rev.* **73**, 1-13 (2013).

30 Estes, R. *Behavioral guide to African mammals: including hoofed mammals, carnivores, primates* (UC Press, Berkeley, Calif, 1991).

31 Bedaso, Z., Wynn, J. G., Alemseged, Z. & Geraads, D. Paleoenvironmental reconstruction of the Asbole fauna (Busidima Formation, Afar, Ethiopia) using stable isotopes. *Geobios* **43**, 165-177 (2010).

32 Bocherens, H., Sandrock, O., Kullmer, O. & Schrenk, F. Hominin palaeoecology in Late Pliocene Malawi: First insights from isotopes (13C, 18O) in mammal teeth. *S. Afr. J. Sci.* **107**, 01-06 (2011).

33 Cerling, T. E., Harris, J. M. & Leakey, M. G. Browsing and grazing in elephants: the isotope record of modern and fossil proboscideans. *Oecologia* **120**, 364-374 (1999).

34 Harris, J. & Cerling, T. Dietary adaptations of extant and Neogene African suids. *J. Zool.* **256**, 45-54 (2002).

35 Van der Merwe, N. J. Isotopic ecology of fossil fauna from Olduvai Gorge at ca 1.8 Ma, compared with modern fauna. *S. Afr. J. Sci.* **109**, 1-14 (2013).

36 Kingston, J. D. & Harrison, T. Isotopic dietary reconstructions of Pliocene herbivores at Laetoli: Implications for early hominin paleoecology. *Palaeogeogr. Palaeocl.* **243**, 272-306 (2007).
